# Intersubjectivity and co-constructed framings: students’ role-play talks in online English-speaking sessions

**DOI:** 10.3389/fpsyg.2024.1499192

**Published:** 2025-01-07

**Authors:** Leslie Huishan Li

**Affiliations:** Department of English, Faculty of Arts and Humanities, University of Macau, Macau SAR, China

**Keywords:** classroom interaction, online learning, student interaction analysis, role-play activity, conversation analysis

## Abstract

This study investigates how lower-level English language learners achieve and maintain intersubjectivity and navigate through co-constructed framings during context-embedded tasks such as improvised role-play. In language education settings, activities associated with interactions often reflect multi-layered participant orientations beyond mere linguistic execution. From the perspectives of ethnomethodology and conversation analysis, linguistic actions are effective only when they are intersubjectively understood: the complexity of an activity necessitates corresponding levels of intersubjectivity for smooth progression. A close examination of sequence development in role-play activities shows how intersubjectivity is preserved as interlocutors engage in aligning and affiliative moves to avoid potential disruptions and maximize mutual contributions. Interlocutors’ progressive inputs are integrated as intersubjectively sustained elements of ongoing interaction framings only when collaboratively developed by the participating parties. The analysis also addresses the impact of online communication tools, acknowledging their increasingly essential role in online teaching. The results suggest the need for a dynamic concept of ‘framing,’ replacing ‘frame,’ and recommend that conversation analysis should account for the multi-layered contexts of surrounding activities.

## 1 Introduction and literature review

### 1.1 CA, English language teaching, and student interaction

Conversation Analysis (CA), with its strength in detailing the nuances of social interaction, has been extensively applied to English language teaching, exploring both the “What” and “How” of the field [see Waring (2019), for an overview]. In defining “What to be taught” in English language teaching (ELT), CA-informed research has significantly contributed to the (re)conceptualization of interactional competence as the primary educational goal [examples include Barraja-Rohan (2011), Hall (2018), and Harumi (2023)]. It has also critically evaluated textbook design, ensuring alignment with real-life communication practices (e.g., Fredagsvik, 2023; Gardner, 2000; Wong, 2007). Additionally, CA research bridges theoretical advancements with practical applications in teaching by closely examining classroom instruction. CA-informed classroom discourse studies have scrutinized teachers’ strategies for managing participation. CA-informed classroom discourse studies have examined teachers’ strategies for managing participation (e.g., Fagan, 2012; Hosoda and Aline, 2013) and delivering instructions (e.g., Gosen et al., 2024; Markee, 2015; Seedhouse, 2008), thus informing “how teaching is conducted.”

Moreover, CA works have demonstrated changes in portraying classroom interactions from teacher-fronted question-answer-comment sets to a nexus of interrelated speech exchange systems (Markee, 2000; Tai, 2023). These changes have been promoted by research on students’ execution of interactional tasks in the classroom or where “learning-in-and-as-interaction” (Koschmann, 2013, p. 2) occurs. Such tasks are often designed in ways that afford the target language for communicative purposes and prompt learners to produce intended interactions (Huth, 2011). CA researchers have then investigated turn-to-turn behaviors and envisioned learning as local and contingent occasions. Previous CA studies discussed how learners collaboratively navigate diverse task trajectories (e.g., Hellermann and Doehler, 2010) and how different task stages as observable social processes become loci of learning (e.g., Markee and Kunitz, 2013; Doehler and Eskildsen, 2022).

### 1.2 CA, role-play, and applications in educational settings

Conversation analysts were among the earliest to investigate role-play interactions (Francis, 1989; Sharrock and Watson, 1985). These studies revealed that role-play is structured by participants’ culturally bound reasoning protocols (Sharrock and Watson, 1985). Recent CA studies recognized that elicited role-play interactions share interactional features as in real-life conversations (Sikveland et al., 2023; Stokoe, 2013). In educational settings, role-play data can be used to examine how social actions are accomplished through learners’ talk-in-interaction. This allows for conducting assessments and analyzing the needs of language learners (Youn, 2020). In ELT settings, role-play is a widely applied task type that requires highly context-dependent performance.

Okada (2010) studied candidates’ performance in role-play tasks during oral language proficiency tests and found that participants display competencies in talk-in-interaction mechanisms (e.g., turn-taking and sequence organization) that closely resemble those found in actual conversations. Informed by task-based pragmatic needs analysis (Youn, 2018), Youn (2020) investigated participants’ organizations of proposal sequences in role-plays designed for speaking assessment. While approving language teachers’ application of role-plays as pedagogical tasks, Youn (2020) also reported relatively abrupt opening turns by lower-level learners, recommending pre-teaching necessary linguistic resources for organizing actions.

### 1.3 Overview and the present study

Previous research has acknowledged the advantages of engaging language learners in classroom activities in which the target language is designed for specific communicative purposes (Dos Santos, 2020; Lee, 2000). Moreover, CA research proved that interactants in role-play activities discursively accomplish social actions and deploy talk-in-interaction mechanisms that are highly similar to those in real-life conversations (see 1.2 above). Despite bearing such potential, existing studies show scarce attention to adopting role-play in language and communication development. Though a refined framework, the Conversation Analytic Role-play Method (CARM) proposed by Stokoe (2014) focused specifically on professional training, such as for language professionals and healthcare providers (Church and Bateman, 2020; Niemants et al., 2023). On learner interactions in ELT, the few existing CA studies analyzed role-play interactions only in oral tests or assessment tasks from the lenses of interactional competence and assessment criteria (Havadar and Balaman, 2024; Youn, 2020).

The present study aims to fill such gaps by examining learners’ role-play conversations in non-assessment-related class activities, focusing on how interactants collaboratively maximize mutual construction and advance the fluent progress of the conversations. While Youn’s (2020) study suggested that role-plays might not be appropriate for lower-level learners due to the requirement for managing contextual performances, findings from this study demonstrate how lower-level English language learners utilize communication resources and successfully maintain intersubjectivity. Data presented in this article are generated from an online English-speaking course designed for lower-level adult learners and are analyzed using conversation analysis. The course was conducted during a pandemic quarantine period through an online meeting platform; hence, the influence of online communication was also examined.

### 1.4 Data collection

The original dataset was generated from a series of online English language courses for lower-level adult learners. The participants consist of lower-intermediate level [A1-A2 CEFR, see British Council (2024)] Chinese learners who participated in weekly grammar and speaking sessions to improve their oral proficiency. In the speaking sessions, the learners engaged in group or pair tasks tailored to elicit peer conversations, thereby practicing the knowledge acquired in corresponding grammar sessions. Most of the tasks encompass topics closely related to the learners’ daily lives and prompt them to simulate their routine social interactions in the target language. The online courses were conducted through an e-platform developed by the course provider. It should be noted that during the role-play activity, the students chose not to turn on their cameras, and only the performing pair would unmute themselves; the instructor also stayed camera-off and muted during each pair’s play process to allow undistracted interaction. Therefore, the target segments generated audio-only pair-talk data. The audio recordings were considered to be of a high standard by colleagues during multiple data sessions. For audio recordings in the dataset, written consent from relevant participants was acquired, all data excerpts were fully de-identified through thorough pseudonymization, and the presented excerpts went through participant member-checking for anonymity.

**Figure 1:**
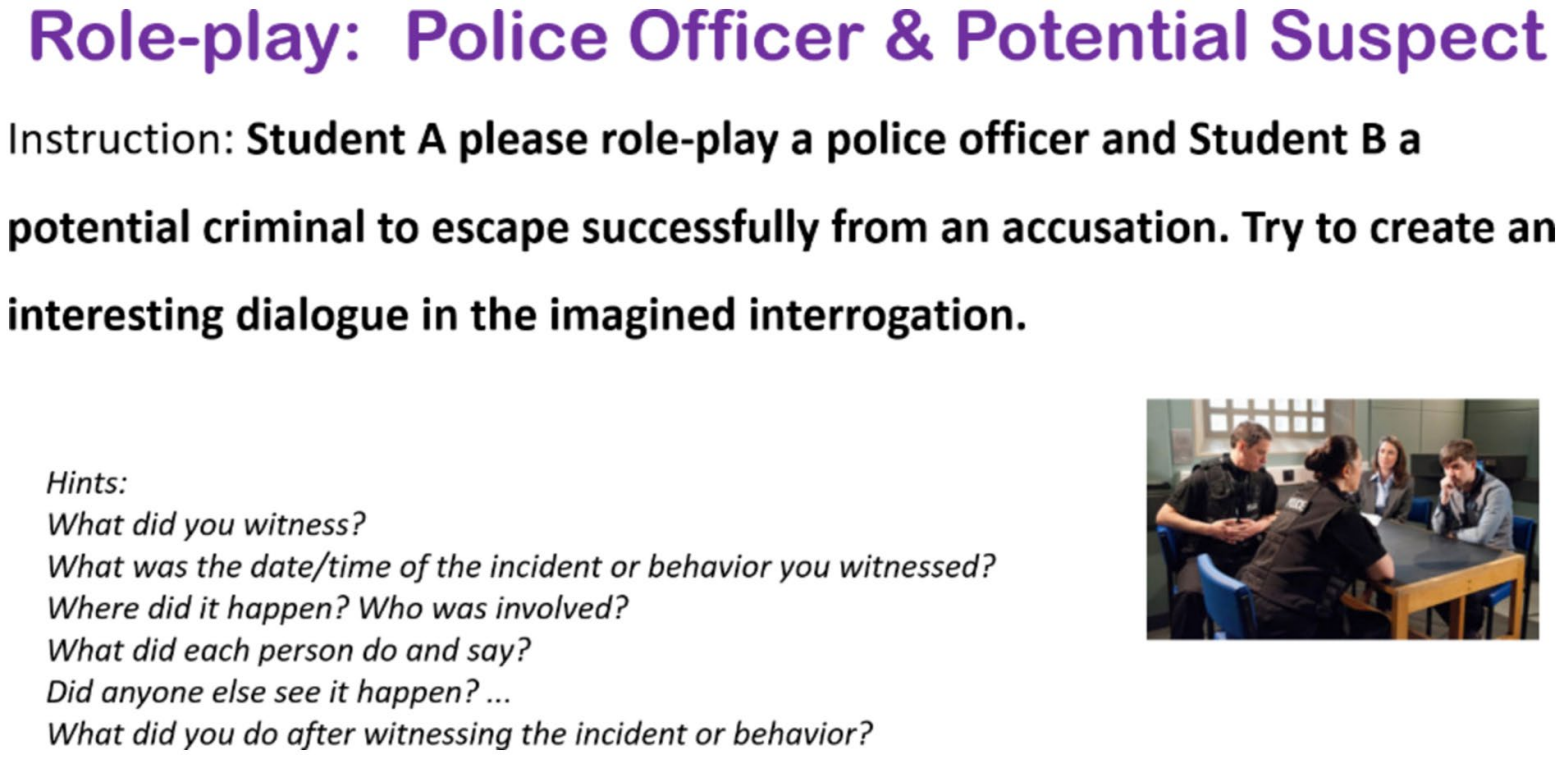
Instruction page1 for the role-play activity.

Data analyzed in this study are drawn from a set of 10-week speaking sessions attended predominantly by junior undergraduate students. The excerpts presented in this article are drawn from three learner pairs’ co-constructed talks in an improvised role-play activity (see Table 1), in which they were asked to role-play a “police officer interrogating potential suspect” scene (see Figure 1 below)^1^. The instructor led the learners through some basic inquire-and-answer expressions taught in the previous grammar session, whereas this activity had no pre-set expressions or plots and aimed to elicit learners’ naturally occurring and unscripted interaction. The learners were given 15 min for discussion and rehearsal, during which the instructor circulated within the classroom to provide assistance when required. Each paired role-play lasted 3–6 min, and the selected learner pairs showed comparatively balanced mutual contribution during the plays.

**Table 1:** Basic information of the selected student pairs.

(Pseudonym initials)	Role division	Main plots	Duration
Pair 1: A and B	A as police, B as a suspect	Alibi debate; witness reference	5 min 40 s
Pair 2: P and J	P as police, J as a suspect	Scene affirming; lawyer reference	3 min 20 s
Pair 3: Z and N	Z as police, N as a suspect	Framing another suspect	3 min 35 s

### 1.5 Data analysis

From the CA perspective, linguistic actions have effects only when intersubjectively understood as so – the more complex an activity becomes, the more intricate intersubjectivity is required for it to proceed (Enfield and Sidnell, 2021). Highly contextual classroom activities such as role-plays could be notably challenging for lower-level learners as they are required to use a work-in-progress language. Previous CA studies on role-play activities in ELT primarily focused on the assessment of learners’ interactional competence and with deficit assumptions on lower-level learners’ performance in organizing such conversations. In contrast, data analysis in this study looks at how lower-level learners maintain mutual intersubjectivity by deploying contextual resources.

Data analysis also reveals the need for and adopts the concept of “framing” to replace “frame” (MacLachlan and Reid, 1994). In these role-play conversations, interlocutors assume dual roles as characters in the role-play and task performers in the language class, engaging with coexisting realms of contextualization, or “frames”: the conversation in which the play occurs, the play activity itself as an in-situ performance, and the played event unfolding in imaginary space and time (Young, 1987). Such coexisting realms are dynamic and contingently constructed by interlocutors’ moment-by-moment collaborative interactions, and there could naturally be several competing frames in the same talk. To analyze such dynamic, ever-changing, and multi-layered contexts and activities in conversations, “framing” is adopted instead of the more or less static notion of frame (Linell and Thunqvist, 2003).

The dataset was transcribed verbatim following Jefferson’s (2004) CA transcription conventions (see Supplementary Appendix A). The transcriptions underwent three rounds of detailed revisions and two rounds of peer review in data sessions with CA researchers. After finalizing the transcription, I conducted a data exploration with unmotivated-looking, maximizing emergent interactional patterns from the data while avoiding deliberately picking out excerpts to fit into pre-determined concepts or frameworks (ten Have, 2007). I first analyzed randomly selected data excerpts to acquire a general picture of interactional mechanism patterns, such as sequence organization, turn-taking, repair, and laughter. This process identified recognizable sequential groups in terms of how intersubjectivity and conversation advancement are collaboratively maintained under online and audio-only conditions. Grasping the general picture of the interactional mechanisms and recognizable sequences allowed me to identify and organize the interlocutors’ representative patterns across events, suggesting the need for a more dynamic concept of framing. Then, a collection of examples of similar phenomena was developed through several rounds of inductive explorations throughout the dataset. Excerpts presented below are further selected based on intelligibility quality and representativeness in elucidating the analysis focuses above.

## 2 Results

As illustrated above, in this role-play activity, students were asked to work in pairs, improvise, and produce an “interrogation” role-play conversation. In the following analysis, the presented excerpts are selected from three student pairs (see Table 1), with one pair (A and B) of particular focus due to data richness and representativeness.

### 2.1 Improvising role-play task online: achieve and maintain intersubjectivity


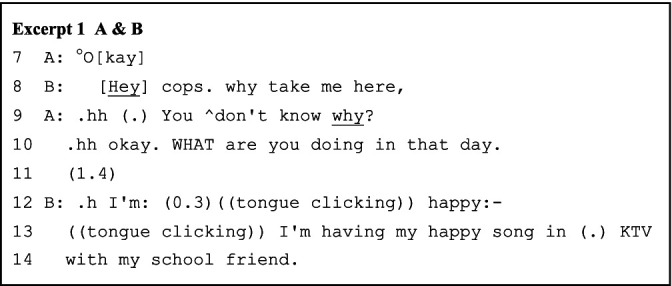


The AB pair’s role-play sequences begin with B’s first turn in line 8, which explicitly draws upon the task prompt (the police-suspect interrogation) and proffers a relevant topic (“Why take me here?”). In the first turn construction unit (henceforth TCU), B’s stress of “Hey” signals an initiation of the upcoming sequences, while “cops” contextually cues A as both the assigned role (the police) and the next speaker. The second TCU then completes the topic with a question, inviting A’s answer to expand the role-play opening upon B’s completed turn. A’s response picks up B’s cue and orients to the projected continuation. A’s rhetorical question (line 9) displays a receipt of both the police officer role and the story expansion expectation while prefacing a stance that B, as the suspect in the role-play story, should provide the alibi statement. The question in line 10 then elicits B’s initiating alibi sequences, which B picks up and plays, following the projected continuation direction again.

As the role-play story unfolds turn by turn, the paired interlocutors adopt multiple strategies to prevent potential conversational problems and sustain the collaborative progression of the role-play sequences. The major strategies include longer pauses, absolute priority of self-repair, and analeptic tying. The lengthy pauses, instead of signaling non-alignment, delay responses to maximize collaborative contribution to the advancement of the talks. For example, in line 11 of Excerpt 1 above, the longer, uninterrupted pause allows B to construct prior-turn-based sequences, with A tacitly cooperating. The interlocutors also confirm the completion of previous turns through longer pauses, considering the absence of nonverbal communicational resources and the possibility of network delay (e.g., line 45 of Excerpt 2/line 12 of Excerpt 3 below). Correspondingly, the struggling self-repairs (line 6 and line 10 of Excerpt 3) are not intervened because the repairing results constitute essential, understandable, and expandable points for the next turn. Further, interlocutors use repetition as an analeptic tying device to invoke earlier-produced elements as resources for achieving interactional work (Mlynář, 2020). As Sacks (1992) noted, the first part of a tying point cannot be identified until the second part is formulated. Here, “KTV” and “sleep more” (lines 39 and 46 of Excerpt 2) are the tying points evoked for both double confirming a transitional element in the previous turn as well as strategically delaying while projecting the upcoming response based on that element. The local past is thus becoming the intersubjectively sustained constituent of ongoing interaction.

Taken together, these ongoing sequences are co-constructed through the interlocutors’ close monitoring of each other’s turn construction, displaying nuanced and in-situ mutual understandings (Enfield and Sidnell, 2021). Because only speech acts can be observed during these online talks, interlocutors purposely adopt pause, repair, and tying strategies to maximize mutual construction. Intersubjectivity is thus achieved and maintained through purposive aligning and affiliative moves as both in-play characters and peer task performers (Stivers, 2008).


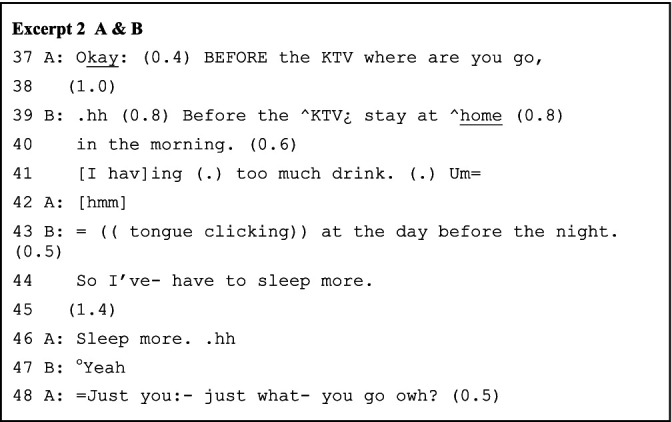



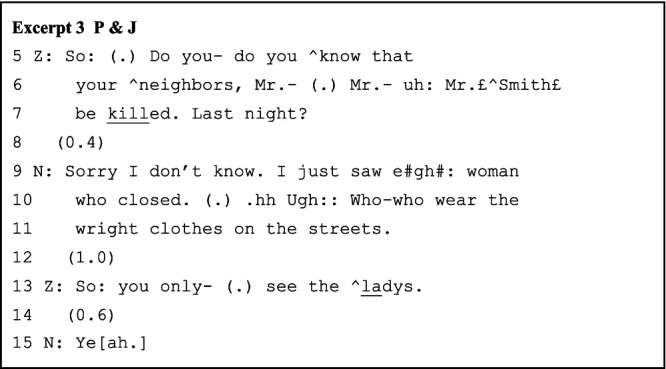


### 2.2 Improvised role-play talks: co-constructed and competing framings

Upon maintaining intersubjectivity, the interlocutors also frame coexisting contexts and perform alongside these ongoing frames-in-frames or competing framings (Linell and Thunqvist, 2003). In Excerpt 1 above, B’s initial turn recognizes A’s epistemic status as the police, thus enacting activity roles and signaling a shift into the specific interaction framing – an interrogation role-play. A’s response, on the one hand, carries on the simulated interrogation framing with prosodic features as the police (lines 9 and 10), whereas on the other hand, it implicitly indicates the playful task framing as A reciprocates the informal appellation “cops” (as opposed to the formal form “sir”).

In Excerpts 4 and 5 above, interlocutors playing the police are to invalidate the suspect’s alibi by introducing and improvising a third party. A’s self-repair from “I” to “we” (line 57 of Excerpt 4) and Z’s “witness” invocation (line 22, Excerpt 5) contribute to the “interrogation” framing by formulating the institutional stance (as “the police”). Moreover, features of troubles “you- uh you guys go- uh > go” (lines 57–60 of Excerpt 4) and repairs “But I hear- I have heard”(lines 21–24 of Excerpt 5) also indicate the ongoing improvised dialog framing where actors have to constantly produce frame-consistent contingencies, as well as the broader learning interaction where learners struggle at language production.

Returning to what happened prior to line 37 in the AB pair’s conversation, on the one hand, the turns are primarily structured as question-and-answer adjacency pairs. On the other hand, the incremental expansions and displayed stances (lines 28, 32, 34 of Excerpt 6 below) based on prior turns indicate how the interlocutors frame the talk with stored realities beyond the ongoing task and the classroom. Such is also how scenes are kept moving in improvised dialogues, in which an actor proposes a new development to the play frame. That proposal becomes a constituent of the ongoing framing only when evaluated and embellished by the other actor. In other words, the actors’ turn-by-turn, co-constructed interactional framings enable and constrain their next actions (Sawyer, 2003).


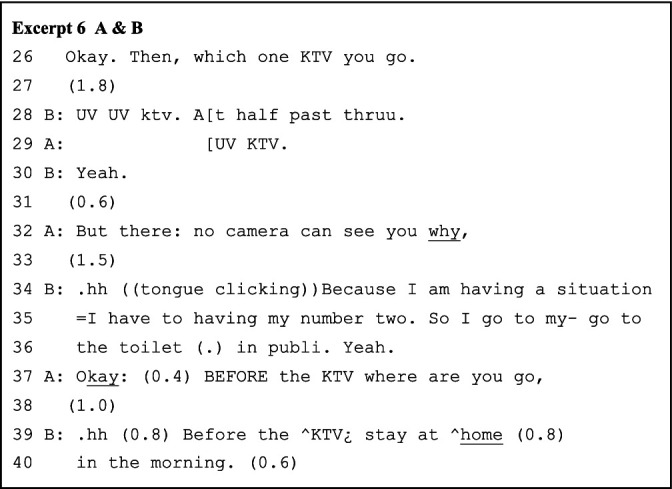


As Goffman (1974) noted, discourse frames depend on participants attending to interactions with stored information from previous experiences. Interaction-advancing orientations are thus both turn-generated as the conversation unfolds and dependent on pre-existing expectations brought by the interactants. While the interlocutors collaboratively co-construct emergent framings through mutually interpretable contextual resources, their expression retrievals are also compatible with the broader framing of their conversation-for-learning and classroom interactions. Conversation sequences are thus simultaneously orienting to and compatible with multiple layers of competing framings. Rather than simply shifting between frames, participants blend or even “embed one within another” through such interdiscursivity (Gordon, 2008, p. 323).

### 2.3 “Moving between” framings: aligning laughter and playfulness

Cases exist, however, in which improvisational players suddenly move out of the ongoing frame and turn to certain metapragmatic actions – what Goffman (1974) referred to as frame-breaking moves. In these instances, discursively marked and abrupt transitions arise, and the players often explicitly deviate from their assigned roles within the primary improvisational framing.

Of Goffman’s particular interest are those frame-breaking moves that are emotionally charged. In Excerpt 7 below, B, the suspect, defends his alibi by delegitimizing the witness’s epistemic status and thus invalidating the statement against him. As discussed earlier, the witness is also an improvised adding element by A, the student, and enacted by A, the police, while it is closely consistent with and expectable following the focal “interrogation” framing. B’s “idiot” utterance (line 62) not only initiates his alibi defense along the focal framing but also noticeably activates the playful task framing, which has also been concomitantly present. This turn is treated by A as a laughable point out of the primarily ongoing interrogation framing. A burst into laughter (line 63) not so much as A the police but more so as A the student or other roles/identities.


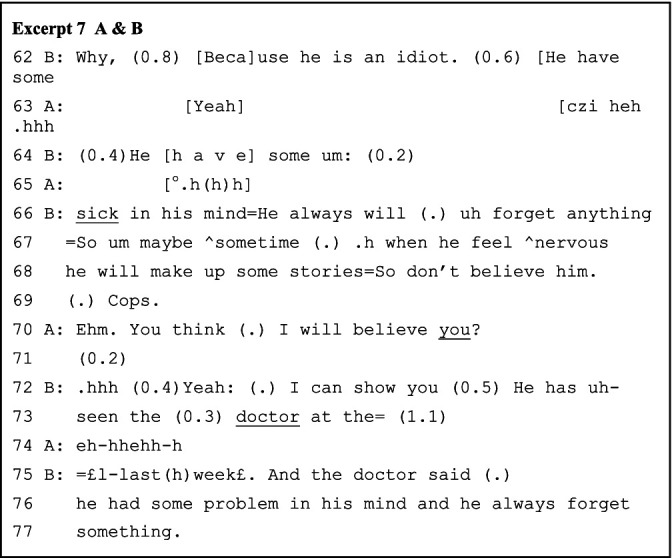


However, laughter requires alignment or appraisal between the participants involved to function properly (Glenn, 2003). In contrast, B only minimally caters to it through short pauses and a stressed voice (lines 64 and 66–69, Excerpt 7 above) before quickly returning to the interrogation framing. Hence, B’s utterances are not specifically designed to make laughter relevant. Although A then attempts to refrain and abandon the laughter (lines 65 and 70) and focus again on the focal framing along B’s commitment, his laughter abruptly reprises (line 74) while B finally joins the laughter (line 75). Therefore, the out-of-frame moves, or what Schegloff (2001) called nonseriousness, extend across multiple turns.

In contrast, N’s out-of-frame laughter (line 39 of Excerpt 8) quickly receives Z’s immediate alignment (line 40), this time with Z attempting to return to the police role and the main focal framing (line 42). However, Z soon joins N in laughter, realigning with the lighter, playful tone. Earlier, in Excerpt 7, A initiates laughter (line 63, Excerpt 7) in overlap with B’s ongoing turn. Here, N, as the current speaker (line 39, Excerpt 8), laughs first, marking her turn with nonseriousness, which shifts its original sequential implications within the interrogation framing. In response, Z’s prosodically inflected “silence” (line 42, Excerpt 8), followed by a short pause, signals an orientation back to reprised playfulness.


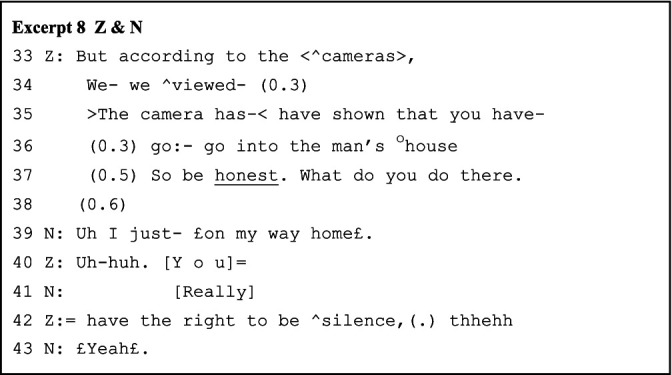


Hence, in these seemingly outer framings, participants tend to behave non-seriously, less consistently, and/or more playfully than in the ongoing focal framings. This is conveyed through contextualization cues such as laughter and aspiration (Gumperz, 1992). Laughter may explicitly frame prior or ongoing turns, and to some extent the upcoming pairs, toward playful ends, thus indicating potential framing boundaries and shifts in behavior. Rather than not carrying the sequential implications within the primary, more serious framing, these turns allow flexible responses as the relevant next along multiple framings (Holt, 2016). Therefore, as discussed in previous sections, participants are, in fact, simultaneously orienting to multiple layers of partially competing yet partially parallel framings.

## 3 Discussion and conclusion

This project investigates how paired English-language learners achieve and maintain intersubjectivity while moving in between co-constructed framings during online role-play talks. A close examination of sequence development made visible how intersubjectivity is meticulously maintained in improvised talks, even by relatively lower-level learners. Interlocutors are oriented toward aligning and affiliative moves to prevent potential problems, maximize mutual contribution, and form coherent interactions. Such moves are notably present as the interlocutors are engaged in online and audio-only communication. As they are only temporally co-present and not physically, the compromised voice delivery (e.g., volume, quality, and network latency) and the absence of non-verbal interpretable communicational resources (e.g., gaze and gestures) naturally alter the procedural infrastructure of interaction. Interactional features such as longer pauses, instant alignment, and inclinations to repair strategies show how collaborative meaning-making orients to the maximization of mutual intersubjectivity and the progressivity of conversations (Moorhouse et al., 2021).

Furthermore, the ongoing sequences in improvised role-play dialogues exhibit the interlocutors’ mutual understanding, which is maintained and evolved moment by moment throughout the conversation. For example, when the responses correspond with previous turns and reflect either alignment or disagreement. In these improvised dialogues, the interlocutors’ progressive contributions are integrated as intersubjectively sustained elements of ongoing interaction frameworks only when collaboratively co-developed by the other party involved. In other words, intersubjectivity serves both as a resource for and an outcome of the interaction (Heritage, 2012), existing as both “chronic” within the temporal scope of the current interaction and “diachronic,” utilizing accumulated experiences and resources accumulated from various contexts (Enfield and Sidnell, 2021; Goodwin, 2018).

The availability of such resources depends on the interlocutors’ ability to bring past experiences into conversations and how these experiences are collectively interpreted and utilized for meaning-making during the interaction. In this study, the interlocutors’ use of communicative resources and strategies emerges from their collective knowledge of, among other factors, interrogation techniques, task-oriented playfulness, and the impact of online communication.

The limitations of this study should be noted. The analysis focuses on audio-only data. Though this may have more accurately explained the turn-by-turn pauses and stresses, the nonverbal, embodied communicational resources (gaze and gesture) normally contributing to social interactions are not included. The audio-only nature of the data also compromised the analysis of the influence across modalities brought by technology as the medium, such as the use of platform functions, the arrangement of online meeting layout, and participant interaction with the interface. In addition, though the analysis presents nuanced meaning-making dynamics, the findings do not comprehensively represent the range of sequence features for this activity type or learner levels due to the sample size. As Walsh (2011) argued, “If we want to look for evidence of learning, we should begin by focusing on the words and interactions of the learner” (p. 188). For language learners, formulating sequenced utterances in coherent and orderly manners is an important step toward advanced proficiency levels (Ortega and Byrnes, 2008).

To conclude, this study proposes CA approaches to examine the turn-by-turn performance of learner conversations and the nuanced forms of meaning-making. First, data analysis and the findings suggest the need for a dynamic concept of framing in replacement of frame to address the ever-changing and emergent nature of interlocutors’ moment-by-moment, co-constructed meaning-making (MacLachlan and Reid, 1994), depicting multi-layered contexts of surrounding parallel activities (Linell and Thunqvist, 2003). Moreover, one important direction for current and future research is to investigate the constraints and affordances of the constantly advancing technological mediums of interpersonal interactions when dealing with naturally occurring conversation data.

Further, this study recommends context-embedded classroom activities such as role-play to help analyze learners’ interactional competencies that are present in actual conversations. For language teachers in particular, observing and facilitating learners’ conversations in context-embedded activities could allow understanding of how collaborative meaning-making and interpretation are achieved with the target language’s varying and often early-stage proficiency. Through deploying a range of communicational resources, learners enact different social and participation roles in the interactions, creating multiple layers to the classroom participation framework and, more importantly, displaying their epistemic knowledge in class (Monfaredi, 2023). Instead of prescriptive and deficit perspectives on task appropriateness or assessing interactional competence for lower-level learners, future studies could take the perspective of resource deployment and negotiation in collaborative meaning-making across different learner levels.

Understanding the accessibility and understandability of resource deployment in meaning-making, or the actual outcome of conversations, would require investigating the differing past trajectories of the parties engaged. While CA approaches primarily focus on the turn-by-turn nuances in talks without making assumptions about broader social settings, studying interactions in specific institutional settings indeed brings attention to the influence brought by wider social and cultural contexts. Future research on language communication and development may consider combining CA with other bottom-up and meticulously descriptive approaches, taking emic perspectives to explore possibilities of mobilizing learner interlocutors’ multifaceted built-in knowledge and resources. This could benefit from cultivating dispositions toward communication-by-repertoire instead of separate language systems and balancing participation dynamics in the classroom and other interactions. Moreover, the differences in personal trajectories and thus unequal access to resources, communicational or others, should be carefully treated in deference to inclusivity and diversity in education.

## Data Availability

The datasets presented in this article are not readily available because the full dataset may include identifiable data. Requests to access the datasets should be directed to the corresponding author leslie.hs.li@connect.um.edu.mo.

## Footnotes

^1^ The animated illustration on the instruction page (Figure 1) is acquired from the open-access and license-free website Freepik.com. https://www.Freepik.com.
