# Supplementary material for: Intersubjectivity and co-constructed framings: students’ role-play talks in online English-speaking sessions
Source: Front Psychol. 2025 Jan 7;15:1499192. doi: 10.3389/fpsyg.2024.1499192 (PMC11747003; doi:10.3389/fpsyg.2024.1499192)
Supplement: Supplementary file 1 [file Image_1.pdf]

Supplementary Material (Appendix A)

Appendix A: Transcription Notation.

SYMBOL	DEFINITION AND USE
[yeah]<br>[okay]	Overlapping talk
=	End of one TCU and beginning of next begin with no gap/pause in between /When TCU continues on new line in transcript.
(.)	Brief interval, usually between 0.08 and 0.2 seconds
(1.4)	Time (in absolute seconds) between end of a word and beginning of next.
Word<br><br>Wo:rd [colon underlined]	Underlining indicates emphasis. Placement indicates which syllable(s) are emphasized.<br>Placement within word may also indicate timing/direction of pitch movement (later underlining may indicate location of pitch movement)
wo::rd	Colon indicates prolonged vowel or consonant.
↑word; ^word<br>↓word	Marked shift in pitch, up (↑) or down (↓). Double arrows can be used with extreme pitch shifts.
.,_¿?	Markers of final pitch direction at TCU boundary:<br>Final falling intonation (.); Slight rising intonation (,)<br>Medium (falling-)rising intonation (¿) (a dip and a rise)<br>Sharp rising intonation (?)
WORD	Upper case indicates syllables or words louder than surrounding speech by the same speaker
°word°; °word	Degree sign indicate syllables or words distinctly quieter than surrounding speech by the same speaker
word-	A dash indicates a cut-off. In phonetic terms this is typically a glottal stop
>word<	Right/left carats indicate increased speaking rate (speeding up)
<word>	Left/right carats indicate decreased speaking rate (slowing down)
.hhh	Inbreath. Three letters indicate 'normal' duration. Longer or shorter inbreaths indicated with fewer or more letters.
hhh	Outbreath. Three letters indicate 'normal' duration. Longer or shorter inbreaths indicated with fewer or more letters.
whhord	Can also indicate aspiration/breathiness if within a word (not laughter)
£word£	Pound sign indicates smiley voice, or suppressed laughter
#word#	Hash sign indicates creaky voice
~word~	Tilde sign indicates shaky voice (as in crying)
(word)	Parentheses indicate uncertain word; no plausible candidate if empty
(( ))	Double parentheses contain analyst comments or descriptions

Adapted from Jefferson (2004); and with reference to Hepburn & Bolden's (2017) *Transcribing aspiration and laughter* on transcription for laughter.
